# Supplementary material for: A set of multi-entry identification keys to African frugivorous flies (Diptera, Tephritidae)
Source: Zookeys. 2014 Jul 24;(428):97–108. doi: 10.3897/zookeys.428.7366 (PMC4143993; doi:10.3897/zookeys.428.7366)
Supplement: Supplementary material 4 — Key to Capparimyia [file zookeys-428-097-s004.zip › SF4_ZooKeys_key to Capparimyia/lucid_player/help/help/whgdata/whlstf4.htm]

Search Words List


**<<**

**node**   
**nodes**   
**nominated**   
**none**   
normal **1** **2**   
**normally**   
note **1** **2** **3** **4** **5** **6** **7** **8** **9** **10** **11** **12** **13** **14** **15**   
notes **1** **2**   
**notification**   
**nt**   
number **1** **2** **3** **4** **5** **6**   
**numbers**   
numeric **1** **2** **3**   
**numerics**   
  
  
objects **1** **2**   
observed **1** **2**   
**obtain**   
**obtaining**   
**off**   
**often**   
**ok**   
**once**   
one **1** **2** **3** **4** **5** **6** **7** **8** **9** **10** **11**   
**ones**   
open **1** **2** **3** **4** **5** **6** **7** **8** **9**   
opened **1** **2** **3**   
**opening**   
opens **1** **2**   
operate **1** **2**   
operating **1** **2** **3**   
**operation**   
operations **1** **2**   
option **1** **2** **3** **4** **5** **6**   
**optionally**   
options **1** **2** **3**   
order **1** **2**   
org **1** **2** **3** **4** **5** **6**   
**organise**   
**organism**   
**organisms**   
**original**   
**os**   
**osx**   
**others**   
**out**   
**outlined**   
**overlaid**   
**overlooked**   
**own**   
**owners**   
  
  
page **1** **2** **3** **4**   
pages **1** **2** **3** **4**   
**palm**   
**palms**   
panel **1** **2** **3**   
panels **1** **2** **3** **4** **5** **6**   
part **1** **2**   
particular **1** **2** **3** **4**   
**particularly**   
**password**   
**paste**   
**pathway**   
paused **1** **2** **3**   
**pentium**   
**people**   
**percentage**   
**performed**   
**perhaps**   
**phoenix**   
**photographs**   
**placed**   
**places**   
plants **1** **2** **3** **4** **5** **6**   
platform **1** **2**   
**play**   
player **1** **2** **3** **4** **5** **6** **7** **8** **9** **10** **11** **12** **13** **14** **15**   
players **1** **2**   
please **1** **2** **3** **4**   
**point**   
**pop**   
**possibilities**   
possible **1** **2** **3**   
**potatoes**   
**powerful**   
**practice**   
**pre**   
preceded **1** **2**   
**preceding**   
**preferences**   
**prefixed**   
present **1** **2**   
**prevent**   
previous **1** **2** **3** **4**   
previously **1** **2**   
**printed**   
**privileges**   
**process**   
**product**   
products **1** **2**   
program **1** **2**   
**programming**   
programs **1** **2**   
progressively **1** **2**   
**prompt**   
**properly**   
provide **1** **2** **3** **4**   
provided **1** **2** **3**   
provides **1** **2** **3** **4** **5**   
**providing**   
prune **1** **2** **3** **4**   
**pruned**   
**pruning**   
**purchased**   
  
  
**quick**   
**quicker**   
**quickly**   
  
  
**ram**   
**random**   
range **1** **2**   
**ranges**   
**rank**   
ranked **1** **2** **3** **4**   
**rare**   
**rather**   
**ratios**   
read **1** **2**   
**real**   
**recalculate**   
recalculated **1** **2**   
receive **1** **2**   
**recently**   
**rechosen**   
recommended **1** **2**   
red **1** **2** **3** **4**   
reduced **1** **2** **3** **4**   
redundant **1** **2**   
redundants **1** **2** **3** **4**   
**reference**   
**referred**   
**refresh**   
**regarded**   
**register**   
**registered**   
**registering**   
**registration**   
related **1** **2**   
**relates**   
released **1** **2** **3**   
remain **1** **2** **3** **4**   
remaining **1** **2** **3** **4** **5** **6** **7** **8** **9**   
**remains**   
**remember**   
remove **1** **2** **3** **4**   
removed **1** **2** **3**   
**removes**   
removing **1** **2**   
reorder **1** **2**   
**reordered**   
**replaced**   
**reports**   
**representing**   
require **1** **2**   
**requirements**   
**requires**   
reset **1** **2**   
**resources**   
**respectively**   
restart **1** **2** **3** **4**   
restarted **1** **2** **3** **4** **5**   
**restarting**   
restore **1** **2**   
**restored**   
**restoring**   
**restrict**   
**restricted**   
result **1** **2**   
**results**   
retain **1** **2**   
**retained**   
**retaining**   
**returning**   
**review**

**>>**
